# Medicine, Pathology and Therapeutics

**Published:** 1897-09

**Authors:** 


### Progress in Medical Science.

### MEDICINE, PATHOLOGY AND THERAPEUTICS.

### A CONTRIBUTION TO THE THERAPEUTICS OF IRON.

GELLHORN, of Berlin, (*Therapeutische Monatshefte,*) says investigations of Quincke have demonstrated incontestably that the favorable results which have been obtained from the administration of iron are actually attributable to its absorption, and not to accidental circumstances, to diet alone, or even suggestion.

At the last Congress for Internal Medicine, the subject of the therapeutics of iron was so thoroughly ventilated by the foremost clinicians, as well as by numerous physicians in late years, that a new contribution would appear superfluous. This subject, however, is of such immense importance to the general practitioner, that accumulation of material is necessary in order to eliminate the least doubt as to the efficacy of a therapeutic measure which, originating at first on the basis of speculation, and later supported by the results of empirical observations, has finally been demonstrated to be of value by exact experimentation.

In the management of chlorosis and anemia, and the host of sequelæ of these diseases, the physician would be powerless if he had not in iron a specific, or at least a potent and indispensable adjunct to his other therapeutic resources. The patients, who belong for the most part to the working classes, give in the main the same group of symptoms; amenorrhea, scanty or profuse, weakening, irregular, usually premature, menses; headache, anorexia and dyspepsia; neuralgias, and almost invariably marked lassitude, which interferes markedly with their ability to work. In these cases prompt and radical help must be afforded, in order to restore to the patients their full working capacity as soon as possible. It is well known that the therapeutic value of the various iron preparations differs greatly. This is shown *a priori* by the abundance of manufactured products of this kind. My experience relates chiefly to three preparations—pilulæ chinini cum ferro, formula magistralis of Berlin, liquor ferri albuminati, and the neutral Pepto-Mangan (Gude). My results with the first of these three remedies have been very indifferent, while with the liquor ferri albuminati of the pharmacopeia they were somewhat better. I have instituted accurate examinations, however, with only Gude's Pepto-Mangan and the data given further on relate to this remedy alone. Owing to my limited experience with the many other preparations employed by various authors, I would not designate the Pepto-Mangan as a universal remedy, or as the only efficient preparation.

In the case of one of my patients I proceeded as follows: I prescribed Pepto-Mangan (Gude), one teaspoonful three times daily after meals, and regulated the diet in accordance with the directions furnished with the preparation. Sour and fatty foods as well as raw fruits, are to be avoided under all circumstances. Fritsch (Diseases of Women, 1892, pp. 469,) advises, indeed, that the desire for acids manifested by chlorotics should be gratified. According to my experience, however, this craving for acids is to be regarded as a pathological condition of the alimentary tract, which is made worse by further supply of acids, but can be successfully overcome by an unstimulating diet. In cases where the social conditions in any way permitted, I allowed the patient to take a small glass of red wine three times daily, but never during a period of one hour before and after the administration of the medicament, in order to prevent the combination of the tannic acid contained in the wine with the iron. The use of potatoes was restricted as much as possible, at least, during the first four weeks. Furthermore, I resorted to the dietetic regulations customary in these cases, but changed them to advantage when, as so often happens, obstinate constipation was present, following in this respect the suggestions of Hebra, which have recently been again advocated by Ruge, (Transactions of the Obstetrical and Gynecological Society of Berlin, 13, III., 1896,) and obtained generally excellent results. In contrast to several authors who made it a practice to remove any existing dyspepsia before resorting to the use of iron, I have followed the method of v. Ziemssen and Baumler, of at once administering iron—unless the presence of a severe gastric affection, especially ulcer of the stomach, could be positively determined—and observed as early as the end of one or two weeks an increase of appetite and subsidence of the gastric disorder.

I would lay especial stress upon systematic exercise in the open air. I ordered the patients who, with but two exceptions, were treated out of bed, to take a stroll at midday, at first of five to ten minutes' duration. At the end of three to four days they were allowed to remain outdoors for five to ten minutes longer.

After each walk they were advised to take off their corsets, put on their slippers and rest for an hour on the sofa. Under this treatment the lassitude invariably vanished after a time.

In the manner thus described I have treated in all about sixty patients. In twenty-four cases I instituted quantitative estimations of hemoglobin at regular intervals of three, five or eight days. Under normal conditions the quantity of hemoglobin in women amounts to 12.59 per cent. when estimated in comparison with the other constituents of the blood. Among my cases the lowest amount met with was in a single instance, 30 per cent. of the normal, that is to say, of the above 12.59 per cent.

I also derived exceedingly favorable results from the use of Pepto-Mangan (Gude) in patients who came to us for operations after being exhausted by protracted hemorrhages. Of course, convalescence in such cases is delayed ; the system recuperates but slowly from the double injury inflicted by the losses of blood and the operative intervention. Digestive disturbances are especially apt to be troublesome. In these cases ferruginous medication often produces remarkable improvement.

I cannot close this paper without calling attention to the beneficial influence exerted by Pepto-Mangan (Gude) in anemic neuralgias.

I think I am justified in asserting that in my therapeutic trials with Pepto-Mangan I obtained all that can be rationally demanded. And I further consider myself warranted in stating that in view of the unquestionable necessity of ferruginous medication in certain troublesome constitutional affections this preparation acts as a most efficient and useful auxiliary to our therapeutic efforts.

---

### THE SPECIFIC ACTION OF QUININE IN MALARIA.

Dr. E. C. Register (Editor of the *Charlotte Med. Jour.*) read a paper with this title (*St. Louis Med. Era*) before the North Carolina Medical Society. After many years of study, both clinical and microscopical, he arrives at the following conclusion in reference to the specific action of quinine in the continued forms of malarial fever : He says a malarial fever without complications will subside after the plasmodia of malaria disappears from the blood ; that we have in quinine the means to completely eradicate malarial poison from the body ; that malarial fever occurring in a previously healthy subject and in the central United States, if at once recognised and properly treated, never ends in death ; that it is speedily curable, never continues, provided the nature of the disease be recognised and appropriate treatment employed.

Register has made microscopical examinations of the blood of several hundred patients suffering with remittent malarial fever, and has studied closely and thoroughly the cresentic and ring-shaped bodies, which he says are the forms of the parasite which is responsible for the continued types of this fever, and he finds that the reason quinine does not always affect these irregular forms of the poison is on account of the usual defects in its administration. He contends that the drug is very imperfectly absorbed when given by the stomach, and when the patient has a temperature of over 102°. He says that in cases of continued malarial fever, if distinct and well-marked intermission of the fever are produced artificially by the use of antipyrine, antifebrine and phenacetine, the cresentic and ring-shaped bodies will disappear after the administration of quinine, as quickly as the spherical bodies that are found in an ordinary case of intermittent fever. In reference to the belief that the forms of the parasite that inhabit the blood-cells are not acted on by quinine, he says : "There is no doubt in my mind that this belief is not erroneous. Besides my own observations I have been able to collect the opinions of thirty-two authors touching upon this point, and twenty-eight out of the thirty-two believe that the endoglobular or intracorpuscular forms are not, on this account, the cause of an uncontrollable fever, and that its proximity to the blood-cell does not, in any way, protect it from the action of quinine."

---

### BRONCHO-PNEUMONIA.

IN THE broncho-pneumonia of children (*Therapeutic Gazette; Louisville Medical Monthly*) when mucus accumulates in the bronchial tubes, and if the child is exceedingly feeble and it is not considered well to administer an emetic, Gaston Lyon orders the following mixture :

| | | |
|---|---|---|
| ℞ | Syrup | 1 ounce. |
| | Syrup of tolu | 1 ounce. |
| | Cognac | 2½ drachms. |
| | Acetate of ammonium | 20 grains. |
| | Benzoate of sodium | 20 grains. M. |

One or two teaspoonfuls of this may be given every hour or every two hours, according to the age of the patient. If the cough is excessive it may be well to add to this mixture from three to four drops of the tincture of belladonna or the tincture of aconite, but, as a general rule, narcotics and antispasmodics are not desirable in very young children.

During convalescence any bronchitis which persists should be treated by the various balsams or by one or two teaspoonfuls a day of a mixture of equal parts of syrup of tolu and syrup of turpentine. In the way of tonics to general nutrition cod-liver oil and hypophosphite of lime are of advantage, and should there be any involvement of the tracheo-bronchial glands or emphysema, iodide of potassium is particularly indicated. Iron is also of value, should anemia be present, prescribed in the dose of three teaspoonfuls a day of the following mixture :

℞ Syrup ........................... 1 ounce.
Syrup of terpine ..................... 2 ounces.
Syrup of iodide of iron ............... 2 ounces.
Peppermint-water .................... 2 ounces.

---

### BRONCHIAL CATARRH.

SÄNGER prescribed, (*Centralblatt für Innere Medicin,*) some six years ago, hydrastis canadensis for a patient suffering with tuberculosis of the larynx and apex catarrh, the immediate occasion of its employment being a trifling hemoptysis. Four days later I found that for three days there had been no blood in the expectoration, and, furthermore, that a tormenting cough with which he had suffered had completely disappeared ; also, the expectoration had become decidedly less and the character of the sputum changed.

---

### FOLLICULAR PHARYNGITIS.

TO BE used (*Clinical Chronicle*) as an application by means of cotton applicator for chronic follicular pharyngitis, post nasal catarrh and atrophic rhinitis :

℞ Iodin. pur ............................ gr. iij.
Potass. iodid .......................... gr. v.
Acid trichloracetic ...................... gr. vij.
Glycerine,
Aq. dest ............................... aa ℥ss.

M. This can be used in varying strengths according to the nature of the case.

---

### INCONTINENCE OF URINE.

℞ Syr. belladonnæ

Syr. tolutani ........................ aa 60.0

Mix. Sig.—Take a coffee-spoonful morning and night.

Or : ℞ Ferri carbon ...................... 0.03 to 0.10
Extr. belladonnæ
Extr. nucis vom.................. aa 0.01 to 0.05

Mix.—Ft. pulv. talcs No. I. (1).

Sig.—Begin with one powder daily, and gradually increase the dose until the physiological effect of the belladonnæ is obtained.

—*Centralb. f. d. Gesam. Theraphie*, 1897, XV., 59.

